# Supplementary material for: Automating General Movements Assessment with quantitative deep learning to facilitate early screening of cerebral palsy
Source: Nat Commun. 2023 Dec 14;14:8294. doi: 10.1038/s41467-023-44141-x (PMC10721621; doi:10.1038/s41467-023-44141-x)
Supplement: Supplementary file 1 — Supplementary Information [file 41467_2023_44141_MOESM1_ESM.pdf]

Supplementary Information

Automating General Movements Assessment with quantitative deep learning for early diagnosis of cerebral palsy

Contents

Supplementary Fig. 1

Supplementary Fig. 2

Supplementary Fig. 3

Supplementary Fig. 4

Supplementary Fig. 5

Supplementary Fig. 6

Supplementary Table 1

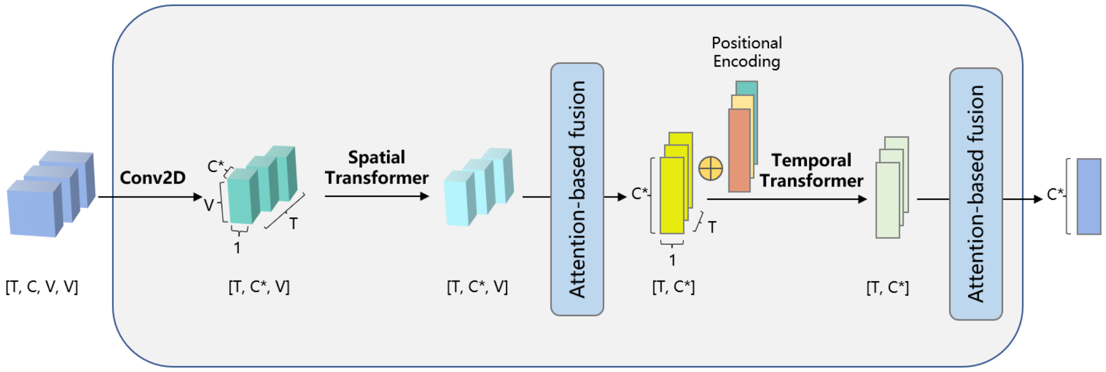

Supplementary Fig. 1. The structure of the spatio-temporal Transformer.

Conv2D (2D convolution) with size $[V, 1]$ fuses the spatial connection information of each joint node into itself. The spatial Transformer and the temporal Transformer exchange information via spatio dimension and temporal dimension respectively. Attention-based fusion integrates and compresses features.

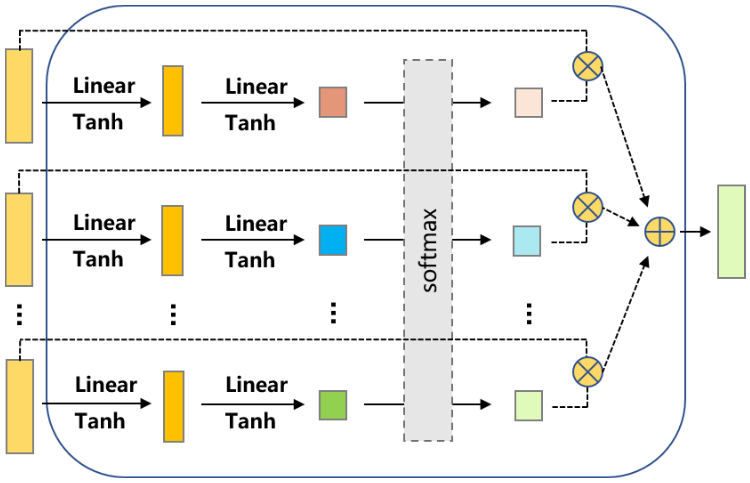

Supplementary Fig. 2. The structure of the attention-based fusion module.

Linear layers and Tanh activation functions give the weight values for input features. The softmax function obtains the normalized weights. Finally, the input feature vectors are linearly combined according to the new weights to create fused output feature.

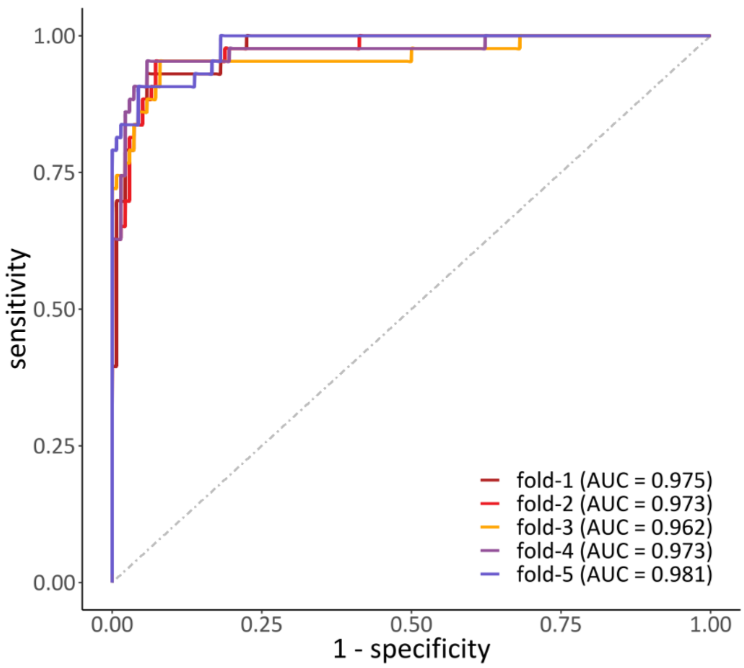

Supplementary Fig. 3. The receiver operating characteristic (ROC) curve of each fold in internal cross-validation.

Each line represents one fold in 5-fold cross-validation. *AUC* area under the ROC curve.

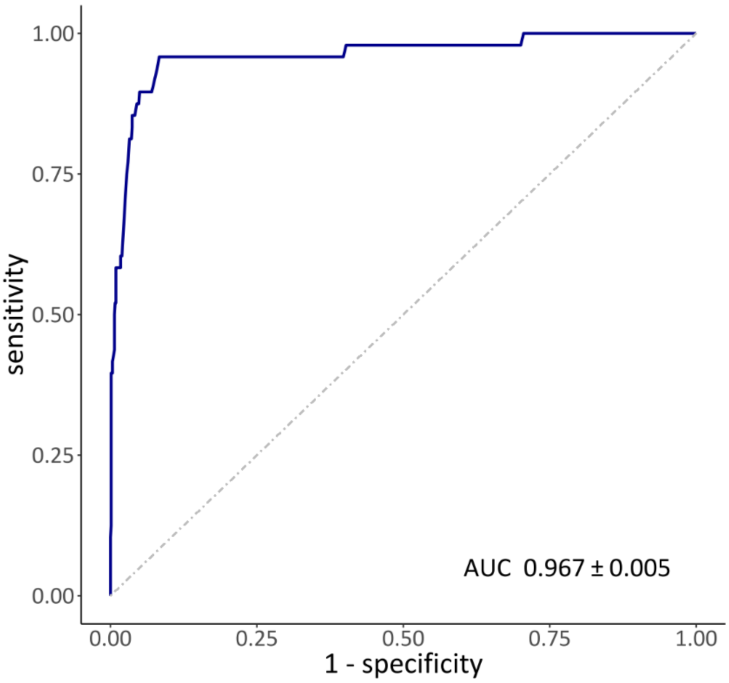

Supplementary Fig. 4. The mean receiver operating characteristic (ROC) curve in external validation.

AUC are represented as mean ± sd. *AUC* area under the ROC curve.

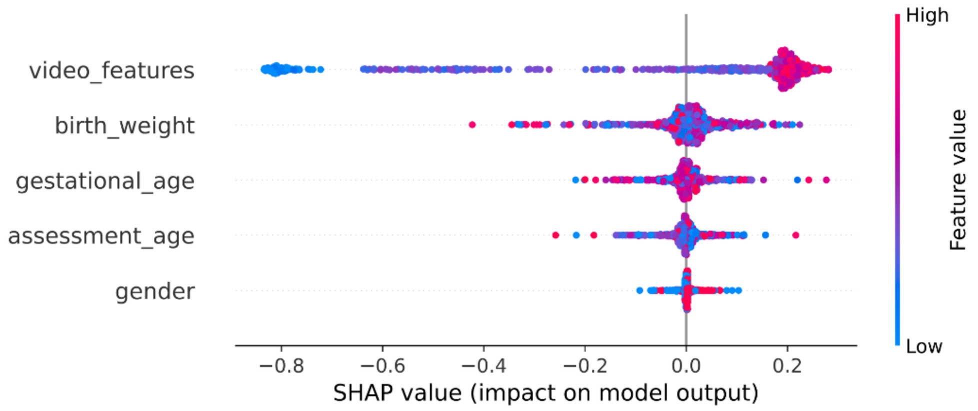

Supplementary Fig. 5. SHapley Additive exPlanations (SHAP) results for input features.

Feature values are indicated by color, with blue for low values and red for high values. SHAP value represents the magnitude of the feature's influence on model output (normal probability), and +/− signs represent the direction of influence.

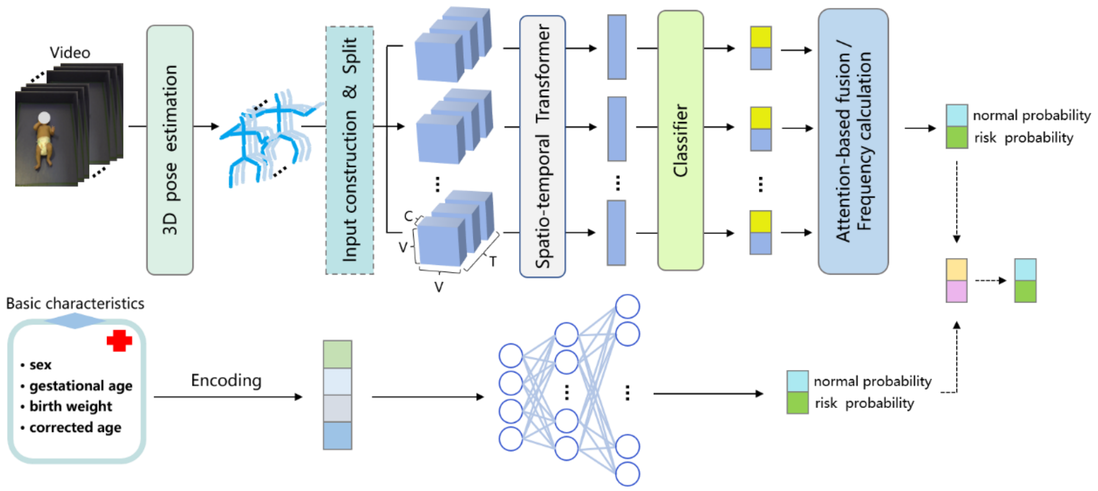

Supplementary Fig. 6. The architecture of MAM in the application.

In the application, the Ref branch is abandoned by MAM. The attention-based fusion step in the Main Branch can be replaced by frequency calculations of FMs clips. Other parts are identical to the original MAM.

Supplemental Table 1. The AUCs under different input features construction ways and pose estimation dimensions.

AUC are represented as mean ± sd. *AUC* area under the receiver operating characteristic curve.

Input construction way	Dimension	AUC
distance matrices	2D	0.943 ± 0.011
distance matrices	3D	0.967 ± 0.005
x, y, z	2D	0.889 ± 0.007
x, y, z	3D	0.900 ± 0.010
x, y, z, v_x, v_y, v_z	2D	0.905 ± 0.013
x, y, z, v_x, v_y, v_z	3D	0.924 ± 0.009
x, y, z, v_x, v_y, v_z, a_x, a_y, a_z	2D	0.923 ± 0.006
x, y, z, v_x, v_y, v_z, a_x, a_y, a_z	3D	0.931 ± 0.006
