# Supplementary material for: Automating General Movements Assessment with quantitative deep learning to facilitate early screening of cerebral palsy
Source: Nat Commun. 2023 Dec 14;14:8294. doi: 10.1038/s41467-023-44141-x (PMC10721621; doi:10.1038/s41467-023-44141-x)
Supplement: Supplementary file 3 — Reporting Summary [file 41467_2023_44141_MOESM3_ESM.pdf]

Corresponding author(s): Guangjun Yu; Hui Lu

Last updated by author(s): Nov 6, 2023

Reporting Summary

Nature Portfolio wishes to improve the reproducibility of the work that we publish. This form provides structure for consistency and transparency in reporting. For further information on Nature Portfolio policies, see our Editorial Policies and the Editorial Policy Checklist.

Statistics

For all statistical analyses, confirm that the following items are present in the figure legend, table legend, main text, or Methods section.

n/a	Confirmed	
☐	☒	The exact sample size (*n*) for each experimental group/condition, given as a discrete number and unit of measurement
☐	☒	A statement on whether measurements were taken from distinct samples or whether the same sample was measured repeatedly
☐	☒	The statistical test(s) used AND whether they are one- or two-sided<br>*Only common tests should be described solely by name; describe more complex techniques in the Methods section.*
☒	☐	A description of all covariates tested
☐	☒	A description of any assumptions or corrections, such as tests of normality and adjustment for multiple comparisons
☐	☒	A full description of the statistical parameters including central tendency (e.g. means) or other basic estimates (e.g. regression coefficient) AND variation (e.g. standard deviation) or associated estimates of uncertainty (e.g. confidence intervals)
☐	☒	For null hypothesis testing, the test statistic (e.g. *F*, *t*, *r*) with confidence intervals, effect sizes, degrees of freedom and *P* value noted<br>*Give P values as exact values whenever suitable.*
☒	☐	For Bayesian analysis, information on the choice of priors and Markov chain Monte Carlo settings
☒	☐	For hierarchical and complex designs, identification of the appropriate level for tests and full reporting of outcomes
☒	☐	Estimates of effect sizes (e.g. Cohen's *d*, Pearson's *r*), indicating how they were calculated

Our web collection on statistics for biologists contains articles on many of the points above.

Software and code

Policy information about availability of computer code

Data collection	The code of HRNet which was used to get the kernel of VideoPose3D is in https://github.com/stefanopini/simple-HRNet.git. The code of VideoPose3D which was used to get the 3D coordinates is in https://github.com/facebookresearch/VideoPose3D.git.
Data analysis	The code for MAM is available in the github repository (https://github.com/qiang-Blazer/MAM). The statistical analyses and tests were down by R packages.

For manuscripts utilizing custom algorithms or software that are central to the research but not yet described in published literature, software must be made available to editors and reviewers. We strongly encourage code deposition in a community repository (e.g. GitHub). See the Nature Portfolio guidelines for submitting code & software for further information.

Data

Policy information about availability of data

All manuscripts must include a data availability statement. This statement should provide the following information, where applicable:

- Accession codes, unique identifiers, or web links for publicly available datasets
- A description of any restrictions on data availability
- For clinical datasets or third party data, please ensure that the statement adheres to our policy

The datasets used in this study are available under restricted access due to privacy, ethical and legal considerations. Access can be obtained by contacting the

corresponding author at gjyu@shchildren.com.cn who will provide a response within 14 days and supply the data use agreement limiting its use to non-commercial research purposes.

Research involving human participants, their data, or biological material

Policy information about studies with human participants or human data. See also policy information about sex, gender (identity/presentation), and sexual orientation and race, ethnicity and racism.

Reporting on sex and gender	The findings of our study apply to both sexes. Biological sex was determined based on parents' reporting and confirmed through physical examination, where applicable. We conducted an analysis to investigate sex differences between the normal and risk groups, as shown in Table 1. Additionally, sex was included as one of the inputs in the Info Branch of the MAM model to assess its predictive value.
Reporting on race, ethnicity, or other socially relevant groupings	No socially relevant categorization variables were used in our study.
Population characteristics	We used sex, gestational age, birth weight, and corrected age of the human research participants. The characteristics differences between the normal and risk groups were assessed, as shown in Table 1. Additionally, all the characteristics were included as inputs in the Info Branch of the MAM model to assess their predictive values.
Recruitment	The participants recruited: from the high-risk infants receiving GMA, the portion of the informed consent signed by the parents was selected. However, it's essential to acknowledge that there might be a potential self-selection bias due to voluntary participation. This self-selection bias could impact the generalizability of the results, as those who chose to participate may differ in certain ways from those who did not.
Ethics oversight	This study was approved by the Ethics Review Committee at Shanghai Children's Hospital, Shanghai Jiao Tong University.

Note that full information on the approval of the study protocol must also be provided in the manuscript.

Field-specific reporting

Please select the one below that is the best fit for your research. If you are not sure, read the appropriate sections before making your selection.

☒ Life sciences ☐ Behavioural & social sciences ☐ Ecological, evolutionary & environmental sciences

For a reference copy of the document with all sections, see nature.com/documents/nr-reporting-summary-flat.pdf

Life sciences study design

All studies must disclose on these points even when the disclosure is negative.

Sample size	In the paper "A Spatio-temporal Attention-based Model for Infant Movement Assessment from Videos": 168 examples for the training set; and 47 examples for the validation set. They got good performance on these sample sizes. In our study, 906 infants were retained in Cohort 1 for 5 fold internal cross-validation, and 221 infants were retained in Cohort 2 for external validation. Far larger than the sample size of the previous study.
Data exclusions	Infants whose corrected ages fall outside the range of 9-20 weeks, infants lacking basic characteristics, or infants whose corresponding videos do not meet the GMA recording requirements have been excluded from Cohort 1 and Cohort 2. Infants with abnormal FMs have also been excluded, as this type is rare and has low predictive power.
Replication	We used 5-fold internal cross-validation, the results were stable in the 5 attempts. And the 5 trained MAM's performances were tested on external validation, which showed good and stable results.
Randomization	In the 5-fold cross-validation, five subdatasets were obtained by stratified sampling by controlling the proportion of people in normal group and risk group.
Blinding	The investigator were blinded to group allocation during data collection and analysis.

Reporting for specific materials, systems and methods

We require information from authors about some types of materials, experimental systems and methods used in many studies. Here, indicate whether each material, system or method listed is relevant to your study. If you are not sure if a list item applies to your research, read the appropriate section before selecting a response.

Materials & experimental systems

n/a	Involved in the study
☒	☐ Antibodies
☒	☐ Eukaryotic cell lines
☒	☐ Palaeontology and archaeology
☒	☐ Animals and other organisms
☒	☐ Clinical data
☒	☐ Dual use research of concern
☒	☐ Plants

Methods

n/a	Involved in the study
☒	☐ ChIP-seq
☒	☐ Flow cytometry
☒	☐ MRI-based neuroimaging
